# Phylogenetic Framework and Biosurfactant Gene Expression Analysis of Marine *Bacillus* spp. of Eastern Coastal Plain of Tamil Nadu

**DOI:** 10.1155/2014/860491

**Published:** 2014-02-12

**Authors:** Sreethar Swaathy, Varadharajan Kavitha, Arockiasamy Sahaya Pravin, Ganesan Sekaran, Asit Baran Mandal, Arumugam Gnanamani

**Affiliations:** ^1^Microbiology Division, CSIR-Central Leather Research Institute, Adyar, Chennai, Tamil Nadu 600 020, India; ^2^Environmental Technology Division, CSIR-Central Leather Research Institute, Adyar, Chennai, Tamil Nadu 600 020, India

## Abstract

The present study emphasizes the diversity assessment of marine *Bacillus* species with special reference to biosurfactant production, respective gene expression, and discrimination among *Bacillus licheniformis* and *Bacillus subtilis*. Among the 200 individual species of eastern coastal plain of Tamil Nadu screened, five biosurfactant producing potential bacterial species with entirely different morphology were selected. Biochemical and 16S rRNA gene sequence analysis suggested that all the said five species belong to *Bacillus* genera but differ in species levels. Biosurfactant of all the five species fluctuates in greater levels with respect to activity as well as to constituents but showed partial similarity to the commercially available surfactin. The expression of *srf* gene was realized in all of the five species. However, the *sfp* gene expression was observed only in three species. In conclusion, both *B. licheniformis* and *B. subtilis* demonstrate *srf* gene; nevertheless, *sfp* gene was expressed only by *Bacillus subtilis*.

## 1. Introduction

Biodiversity is given by a variety of species living on earth resulting from billions of years of evolution. Molecular-phylogenetic studies have revealed that the main diversity of life is microbial and it is distributed among three domains: prokaryotes, eukaryotes, and archaea. The functioning the of whole biosphere depends absolutely on the activities of the microbial world. Due to versatility, microbes are the major natural providers of ecological services as well as playing major role in semiartificial systems such as sewage treatment plants, landfills, and in toxic waste bioremediation. However, because of the continuous exposure to man-made synthetic molecules a large change/reduction in microbial diversity has been realized. Several publications document the effect of chemical pollutants on microbial community structure based on the available sequencing technologies [1]. Metagenomics, metatranscriptomics, metaproteomics, and single-cell sequencing are the approaches providing a view not only of the community structure (species phylogeny, richness, and distribution) but also of the functional (metabolic) potential of a community.

Among the various sources, microbes of marine waters/sludges received good attention because of the wide potential biological products with immense applications [2]. Most of the products are used to recover oil [3–5]. In addition, the interest in marine bacteria arises from their biotechnological potential in bioremediation [6] and in oceanic biogeochemical cycles [7] and as a source of novel metabolites different from those isolated from terrestrial bacteria, such as antibiotics [8] and products for industrial use like exopolysaccharides, enzymes [9], biosurfactants, and compatible solutes [10]. Most of the available investigations focused on the two groups of bacteria, the members of the genera *Bacillus* or *Pseudomonas*, due to the resourcefulness and applications [5, 6, 11–15]. With regard to *Bacillus* genera, it consists of more than 222 recognized species (http://www.bacterio.cict.fr) distributed widely across many terrestrial and aquatic habitats [15, 16] including marine sediments [17]. Members of the genus *Bacillus* comprise Gram positive, spore forming, rod-shaped, and aerobic bacteria. *Bacillus* species are phenotypically and genotypically heterogenous [18]. Few studies have focused on the phylogeny and biodiversity of marine Bacilli [15–17, 19]. These reports were based on a small number of isolates or a unique sampling site and showed that *Bacillus subtilis, Bacillus licheniformis, Bacillus cereus, Bacillus amyloliquefaciens,* and *Bacillus pumilus* are the common inhabitants of marine environments and consequently often isolated. However, a more extensive analysis to assess the real biodiversity and phylogenetic relationship between Bacilli from marine origin is becoming necessary. Recent studies on marine bacilli showed that strains of *B. marinus, B. subtilis, B. pumilus, B. licheniformis, B. cereus, *and* B. mycoides* are common inhabitants of the marine habitat [15]. They constitute closely related taxa and their differentiation has become difficult and laborious, as they cannot be distinguished from each other by phenotypic tests.

Furthermore, with regard to biosurfactant production, certain strains of the closely related species *B. subtilis, B. mojavensis, B. sonorensis, *and* B. licheniformis* produce the cyclic lipopeptide biosurfactants, surfactin, and lichenysin [20–23]. *B. subtilis* produces a known lipopeptide biosurfactant called surfactin, which is coded by four open reading frames (ORFs) named as SrfA, SrfB, SrfC, and SrfD [24]. Similarly, lichenysin is a lipopeptide biosurfactant produced by *B. licheniformis* coded by lichenysin operon (LchA) and consists of three peptide synthetase genes: LicAA, LicAB, LicAC, and LicAD [21]. *B. cereus* produces a lipopeptide biosurfactant called plipastatin [25].

The species most closely related to *B. subtilis* are unusually similar at the phenotypic level. For example, fatty acid composition is the only known phenotypic character that distinguishes *Bacillus mojavensis* and *Bacillus vallismortis* from one another or from *B. subtilis* [26, 27] and *Bacillus atrophaeus* is distinguishable from *B. subtilis* only by differences in pigmentation [28]. A heterogeneous group of moderately halophilic bacteria, which comprises *Bacillus salexigens*, and three species of the new genus *Halobacillus, H. halophilus, H. litoralis,* and *H. trueperi* [29–31], may be differentiated by their ability to grow at 10 to 20% of total salts and the possession of an unusual type of murein. Molecular methods have proven to be more effective in distinguishing close relatives of *B. subtilis*.

From the above-summarized information, it has been understood that discrimination of the *Bacillus* species based on the morphology, phylogenetic framework and gene expression of molecule of interest is possible; nevertheless, the closely related species with similarity in the byproducts need intensive explorations.

Thus, exploring the phylogenetic markers such as 16S rRNA genes to reveal microbial diversity and further exploration of gene expression to reveal the functional power of the microbes is essential. Hence, in the present study, phylogenetic framework of marine *Bacillus* spp. and expression of gene responsible for biosurfactant production has been explored in detail, which provided interesting information and highly relevant to identification of novel strains with desirable functional characteristics and biotechnological applications.

## 2. Materials and Methods

### 2.1. Isolation and Maintenance of Organism

**Figure 1:**
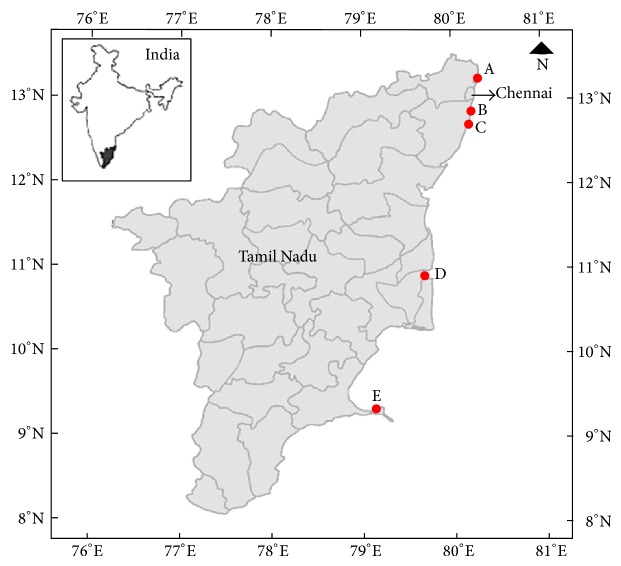
Location of the five sampling sites. Site coordinates limits (marine samples of water, sediments, mussels, shells, sea weeds, and sand): A is Ennore, Chennai, Tamilnadu; B is Mahabalipuram, Tamilnadu; C is Kalpakkam township, Tamilnadu; D is Cuddalore, Tamilnadu; E is Mandapam, Ramanathapuram, Tamilnadu.

Marine samples of water, sediments, mussels, shells, sea weeds, and sand were collected from Kalpakkam, Ennore port, Besant Nagar Beach, Marina beach, Mahabalipuram beach, Mandapam, Vedaranyam, Tuticorin, Cuddalore in Tamil Nadu, India (Figure 1). Zobell marine broth/agar (for bacteria) was the media used for the isolation of microbial species according to the standard procedure employed for the isolation and maintenance of marine organisms. Morphologically distinct organisms were selected and stored in 30% glycerol stock at −20°C.

### 2.2. Screening of Biosurfactant Producing Microbes

Biosurfactant producers were screened based on the surfactant activity exhibited by the culture media after 24–72 hours of incubation at 37°C in the presence of the bacterial isolates. In brief, Zobell marine broth (Hi Media) (100 mL in 1000 mL capacity conical flask) was sterilized and inoculated with the isolated cultures and was incubated at 37°C for 24–72 hours under shaking condition. Followed by incubation, the samples were made cell-free by centrifugation at 10,000 × g at 4°C. Biosurfactants activity of the cell free medium was assessed according to the procedure summarized in the following paragraphs and the isolates exhibiting appreciable surfactant activity were selected and subjected to identification and further production.

### 2.3. Morphological, Physiological and Biochemical Characteristics of Marine Isolates

The strains isolated from marine sediments were identified by conventional biochemical tests in accordance with Bergey's Manual of Systematic Bacteriology [32].

### 2.4. *DNA *Extraction

Genomic DNA was isolated and purified by “Dneasy Tissue Kit” (Qiagen GmgH, Hilden, Germany) according to the manufacturer's directions. The quality of the extracted DNA was checked by agarose gel electrophoresis 2% (w/v).

### 2.5. PCR Amplification: 16S rRNA Gene Sequencing

To confirm the identities of the isolates, PCR amplification and sequencing of the 16S rRNA gene were performed. The 16S rRNA genes (1,500 bp) were purified using the gel elution kit (Sigma-Aldrich, USA) as per the manufacturer's protocol using the bacterial universal primer set of 8F: 5′-AGAGTTTGATCCTGGCTCAG-3′ and 1492R: 5′-GGCTACCTTGTTACGACTT-3′ as described by Turner et al. [33]. Amplification was carried out with the eppendorf thermal cycler (Eppendorf North America Inc.) in a total volume of 25 *μ*L containing about 50 ng of genomic DNA, 5 U of Taq DNA polymerase, 20 pmol of each primer, 200 *μ*M dNTPs, and 1X Taq buffer with 1.5 mM MgCl_2_ according to the following program: 93°C for 2 min, 35 cycles of 93°C for 1 min, 52°C for 2 min, 72°C for 2 min, and final extension at 72°C for 5 min. PCR products were analyzed by electrophoresis in 1% agarose.

### 2.6. Phylogenetic Analysis

For phylogenetic identification, the acquired sequences were used for a gene homology search with the 16S rRNA gene sequences available in the public databases obtained from GenBank using the BLAST program (http://blast.ncbi.nlm.nih.gov/) [34] and ribosomal database project (http://rdp.cme.msu.edu/seqmatch/seqmatch_intro.jsp) [35]. Sequences retrieved from the GenBank database were aligned using the CLUSTAL omega [36], and tree was performed using three tree-making algorithms: neighbor-joining using Tamura-Nei distance parameters in the Geneious R6 program (available from http://www.geneious.com/).

### 2.7. Experimental Setup and Extraction of Surface Active Agents

All the selected isolates were cultured individually in 1000 mL Erlenmeyer flask containing 100 mL of Zobell marine broth and incubated at 37°C for 24–48 hours under shaking condition. Followed by incubation, the cell-free supernatant was subjected to (equal amount of ice cold) ethanol precipitation according to Vater et al. [37]. The residual pellet was obtained upon centrifugation, dissolved in water, subjected to surface activity measurements, and designated as “*microsurf*”.

### 2.8. Surface Activity Measurements

#### 2.8.1. Drop Collapse Test

It is a rapid and crude method to assess the surfactant activity according to Jain et al. [38]. In brief, about 10 mL of cell free broth was added in the center of an oil drop (20 *μ*L of any oil) taken in a clean glass slide. The collapse of oil drop has been visualized and the less time taken indicates the higher activity of a surfactant. Activity of microbial surfactant was compared with water, synthetic (Tween 80, SDS, CTAB) and commercially available biosurfactant (Surfactin).

#### 2.8.2. Oil Displacement Method

This method is so sensitive and only a small amount of sample was required to measure the surfactant activity. About 10 mL of cell-free broth was placed in the center of oil (50 *μ*L of any oil), which was formed on the surface of water in a large size petridish (15 cm diameter). The size of the resultant oil-displaced circle area reflects the activity of a surfactant. The larger the size, the higher the activity of a surfactant [39]. A 50 *μ*L distilled water was used for negative control and 50 *μ*L of 0.1% Triton X 100 was used for positive control experiments.

#### 2.8.3. Tensiometer Measurements

Surface tension of biosurfactant was measured by plate method using GBX-3S tensiometer (DM) at room temperature at different dilutions ranging from 1- to 10-fold [40]. A 10 mL of the cell free broth was taken in a clean glass beaker (20 mL) and was placed on tensiometer platform. A sterile plate was submerged into a solution and then slowly pulled through the liquid-air interface. Stabilization was allowed to occur until a standard deviation of 10 successive measurements was <0.4 mN/m. Each result was the average of 10 determinations after stabilization.

### 2.9. Assessment of Emulsifying Activity, Emulsification Index, and Stability of Emulsion of “Microsurf (1 to 5).”

Followed by the measurement of biosurfactant activity, bioemulsification activity was determined using heptadecane (hydrocarbon) as substrate. In brief, we mixed 50 *μ*L of the sample (*Microsurf* (1 to 5)) with 1.0 mL of sodium acetate buffer (pH 3.0), then added 2 *μ*L of heptadecane and vortex. After 2-3 min, absorbance was measured at 540 nm using UV-visible spectrophotometer (Shimadzu, Japan). One unit of emulsifying activity was defined as the amount of biosurfactant that affected an emulsion with an absorbance at 540 nm of 1.0. To assess the emulsification index, 1.0 ml of cell free broth was mixed with 1.0 ml of various hydrocarbons (oils of sesame, peanut, sunflower, soybean, rice bran, and crude), benzene, toluene, and kerosene individually, vortex for 30 min and kept at room temperature. Emulsification index (E24) was calculated after 24 hours, by measuring the height of emulsion layer with respect to original volume.

For the assessment of stability of emulsion, emulsions obtained from above experiments were kept at room temperature for the period of 0–100 days. Visual observations were made for any phase separation such as, flocculation, creamy, and coalescence. Samples exhibiting nil phase separation were considered as stable emulsion.

### 2.10. Hemolytic Activity on Human Erythrocytes

To assess the hemolytic activity of “*microsurf (1 to 5)*” obtained from the above experiments, human RBC cells were used as substrates [41]. RBC suspension was incubated in the presence of isotonic solution of biosurfactants solution at room temperature for 10 min in the dark. The amount of hemoglobin released upon centrifugation (3000 ×g) was measured at 540 nm. RBC lysis with water was taken as 100%, and sodium dodecyl sulfate (SDS) as reference compound.

### 2.11. Antimicrobial Activity

Antimicrobial activity of the “*microsurf (1 to 5)*” was made according to the CLSI [42] Standards for Antimicrobial Susceptibility Testing, using Gram positive and Gram negative species obtained from Microbial Type Culture Collection (MTCC), Chandigarh. In detail, Mueller-Hinton agar (MHA) was prepared from a commercially available dehydrated base according to the manufacturer's instructions. Followed by sterilization the media was poured in a glass petridishes. The agar medium was then allowed to cool to room temperature. For antimicrobial activity of “*microsurf (1 to 5)*,” 50 *μ*L of (0.5 Mcfarland OD of MTCC strains) MTCC strains spread over the MHA plate and 0.5 mm well was made on the plate and different dilution of *microsurf *(1 to 5) was poured in the well and incubated at 37°C.

### 2.12. Surfactant Gene (*srf*/*sfp*) Expression Analysis

Followed by identification and “*microsurf*” production, the gene of interest was designed from earlier reports [43] and studied according to the standard procedures. The primer pair *srfA3/licA3F*: CAAAAKCGCAKCATATGAG and *srfA3/licA3R*: AGCGGCAYATATTGATGCGGYTC was designed to amplify a 268 bp portion of the *srfA3* or the homologous *licA3* gene present in surfactin/lichenysin-producing strains of the *B. subtilis/B. licheniformis*. These primers were tested against five *Bacillus* strains. The *srfA3/licA3* PCR reaction mix in a volume of 25 *μ*L reaction mixture consists of 2.5 U of Taq DNA polymerase, 100 ng of genomic DNA, 20 pmol of each primer, 200 *μ*M dNTPs, and 1X Taq buffer with 2 mM MgCl_2_. The thermal cycler was programmed for 2 min at 93°C for an initial denaturation cycle, 38 cycles of 35 s denaturation at 93°C, 35 s annealing at 48°C, 45 s extension at 72°C, and followed by a 5 min final extension at 72°C.

With reference to *sfp* gene (the second gene essential to the production of surfactin) [44–46], PCR amplification was studied for all the five isolates with primer pairs specific to the *sfp* gene. The *sfp* 675 bp fragment, corresponding to the *B. subtilis sfp* gene (GenBank accession number X63158) at positions 167–841, was amplified from the genomic DNA by PCR, using two oligonucleotide primers: *sfp* F: (5′-ATGAAGATTTACGGAATTTA-3′) and *sfp*-R: (5′-TTATAAAAGCTCTTCGTACG-3′). Amplification was carried out with eppendorf thermal cycler (Eppendorf North America Inc.) in a total volume of 25 *μ*L containing about 200 *μ*M dNTPs, 1X Taq buffer, 50 ng of genomic DNA, 20 pmol of each primer, and 3 U Taq DNA polymerase. The thermal cycler was programmed for 1 min at 94°C for an initial denaturation cycle, 25 cycles of 1 min denaturation at 94°C, 30 s annealing at 46°C, 1 min extension at 72°C, and followed by a 10 min final extension at 72°C.

PCR products were analyzed by electrophoresis on 1% agarose. The expected DNA bands of *srf/sfp* 268/675 bp were excised from gel and purified using the gel elution kit (Sigma-Aldrich, USA) as per the manufacturer's protocol. The sequencing reactions were carried out for both the gene using a Big Dye Terminator Cycle Sequencing Kit (Applied Biosystems, Foster City, CA, USA). The sequences thus obtained were assembled and edited using Geneious R6.

### 2.13. DNA Sequence Accession Numbers

Accession numbers of sequences determined in this study were deposited in Genbank as isolate 1, HM145910, isolate 2, HM194725, isolate 3, HM222944, isolates 4 and 5, sequences submitted to NCBI.

## 3. Results and Discussion

### 3.1. The Marine Samples of Eastern Coastal Plain of Tamil Nadu

The marine samples collected from eastern coastal plain of Tamil Nadu were subjected to the microbial profile with respect to biosurfactant production which implied that only 9.0% of the bacterial have population shown biosurfactant production. However, the remaining populations displayed secondary metabolites other than biosurfactants. It could be reasoned to the less oil pollution but with greater pharmaceutical waste contaminations. Even the samples of Ennore port and Tuticorin port also showed less biosurfactant producers. It can be argued that the sampling seasons may play the role in the specific microbial populations. However, to avoid this argument, sampling was done at three different seasons and in all the scheduled time periods, only less number of biosurfactant producers were encountered. But, still, the sampling studies need much more explorations.

### 3.2. Phenotypic and Biochemical Characteristics

**Figure 2:**
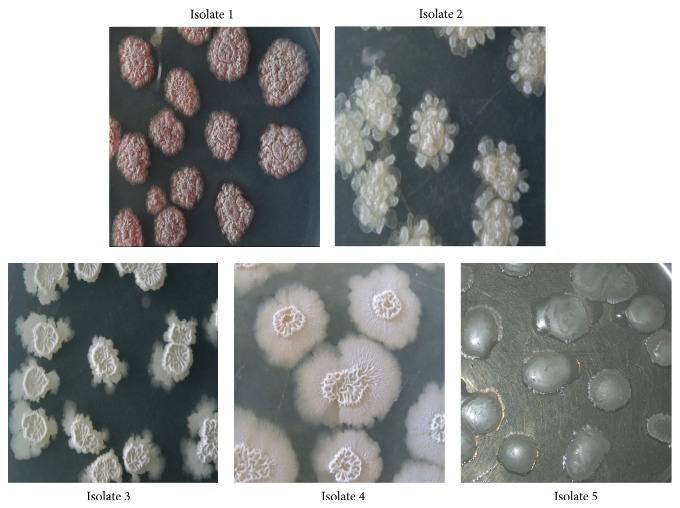
Plate morphology of the five different marine isolates (isolate-1; isolate-2; isolate-3; isolate-4; isolate-5).

Screening and identifications studies revealed that the selected isolates were confirmed as the members of the genus *Bacillus* phenotypically and genotypically. With respect to phenotypic characteristics, most of the colonies were smooth, white in colour, and having regular margin and some of the microbes have rough, dry, irregular margin; and some have slimy with smooth colonies and one isolate exhibited filamentous growth with pigment production on agar plate (Figure 2). Though most of the reports on *Bacillus* species infer nil pigmentation, however, the pigmented colonies of one of the isolates observed in the present study correlate well with the report of Palmisano et al. [47]. With reference to Gram staining, the isolates were Gram positive rod-shaped and dominated in the size range of 0.5–1.2 *μ*m. All the isolates were motile and spore producers.

Biochemical analysis revealed that all five isolates were positive for catalase and one isolate showed positive for oxidase. All five isolates showed hydrolysis of casein, gelatin, starch, and lipid. Few isolates were not responsive to the IMViC test.  Table 1 describes the results on the biochemical analysis of the biosurfactant producers.

### 3.3. Molecular Characterization

**Figure 3:**
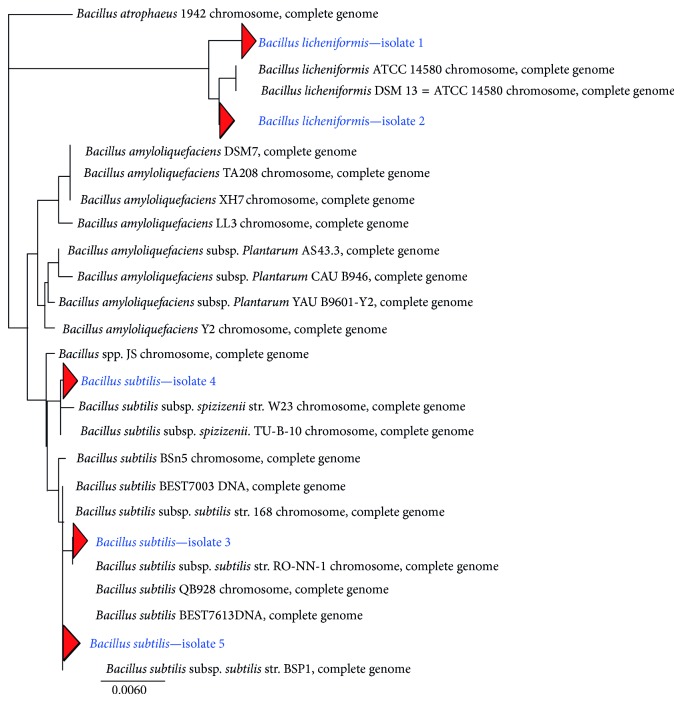
Phylogenetic tree of 16S rRNA gene sequences of the marine isolates. Bar, 0.006 substitutions per nucleotide position.

Results on phenotypic characteristics and 16S rRNA gene sequence homology suggested that all the five isolates have shown close relativeness to genus *Bacillus *(Table 2). Isolates 1 and 2 share an almost identical 16S rRNA gene sequence with the published species *B. licheniformis* ATCC 14580 and* B. licheniformis* DSM13 = ATCC 14580 (94% and 99% similarity), whereas isolates 3, 4, and 5 share an almost identical 16S rRNA gene sequence with the published species *B. subtilis* RO-NN-1 chromosome, *Bacillus* spp. JS chromosome, and *B. subtilis* BEST7613 DNA with sequence homology (100% similarity). The phylogenetic framework was constructed based on the sequence analysis constructed by neighbor-joining method based on 16S rRNA gene sequence (Figure 3). It has been understood that all five isolates are closely related to *B. subtilis* with meager variations. Results of Roberts et al. [26, 27], and Nakamura [28] also observed the similar relativeness in *B. subtilis* with other *Bacillus* spp.

With respect to biosurfactant activity of the isolates, the cell-free broth was subjected to drops collapse test, oil displacement test, and tensiometer measurements. The biosurfactant activity was compared with commercially available biosurfactant, synthetic surfactant, and water. All the isolates collapse the oil but differ at the time taken for collapsing the oil with respect to organism.  Table 3 describes results on the drops collapse test and oil displacement test, and tensiometer measurements suggested that all the five marine isolates exhibit appreciable biosurfactants activity which was on par with synthetic surface active agents, and Table 4 illustrates the chemical composition of biosurfactant obtained from marine isolates. Followed by the production, recovery of biosurfactant by precipitation processes was carried out. Ethanol precipitated biosurfactant (designated as “*microsurf (1 to 5)* respective to isolate 1 to isolate 5”) was creamy white in colour and soluble in water and dimethyl sulfoxide.

Examination of emulsification activity, emulsification index and stability of emulsion reveal, emulsification index of 85–95% was observed with vegetable oils and crude oil and <75% with kerosene. With regard to the stability of emulsions, stable emulsion was observed with vegetable oils (more than 90 days) more than emulsion formed with kerosene (only up to 30 days) and crude oil. Followed by the emulsion formation, we observed a complete transformation of emulsion to thread like structures in crude oil and reasons to less stability. With reference to hemolytic activity, no haemolysis was exhibited by the “*microsurf (1 to 5)*” even at high concentration. The nonhemolytic behaviour of “*microsurf (1 to 5)*” in comparison with standard surfactin observed in the present study may be due to the nonionic nature of the “*microsurf.*” The reason for the nonionic nature has not been understood yet. However, according to the literatures, the presence of aminoacid determines the ionic characteristic of biosurfactant. And moreover, the presence of alanine may also contribute to the nonhemolytic behaviour [48]. Further, the nonantimicrobial activity of “*microsurf*” has also been reasoned to the nonionic nature.

### 3.4. Identification of Gene of Interest and Sequence Determination of PCR Amplified Product

**Figure 4:**
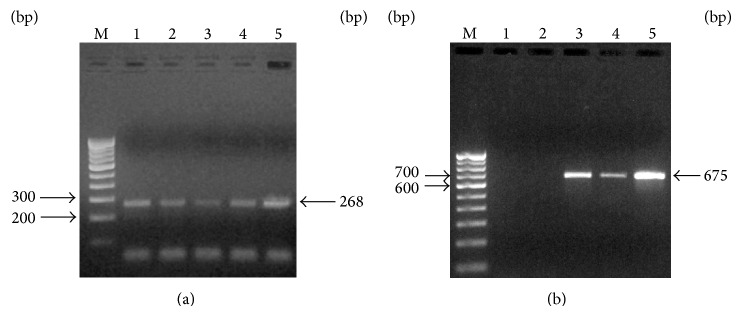
PCR amplification of *srf* (a) and *sfp *(b) gene in the chosen marine isolates. [M-molecular size marker (100 bp ladder); Lane 1 is* B. licheniformis* (isolate 1); Lane 2 is* B. licheniformis* (isolate 2); Lane 3 is* B. subtilis *(isolate 3); Lane 4 is* B. subtilis* (isolate 4), and Lane 5 is* B. subtilis* (isolate 5)].

Figure 4(a) illustrates the PCR amplification of *srf* genes in all the five isolates. It has been observed that all of the five isolates express *srf* gene amplification at 268 bp. However, the expression of *sfp* gene amplified at 675 bp was realized only for three isolates (Figure 4(b)). The *sfp *gene is an essential component of peptide synthesis systems and also plays a role in the regulation of surfactin biosynthesis gene expression [42]. The sequences *srf*/*sfp* thus obtained were verified in the NCBI databases for the gene/species confirmation, thus validating the presence of the genes in the selected strains of *Bacillus*.

According to the existing literatures, discrimination of *Bacillus* species was assessed based on the (i) morphology; (ii) biochemical; (iii) genes responsible for the particular metabolite production/or molecular profile; and finally (iv) the structural features of the metabolites/enzymes. Nevertheless, still, the variation at subspecies levels has been realized by a number of researchers. In the present study, discrimination in *Bacillus* species was made based on the biosurfactant production. Since there was no clear report on the actual steps involved in the synthesis of surface active agents by *Bacillus* species and numbers of hypotheses have been explored by the researchers. The entire said hypothesis as a whole suggested that biosurfactant produced has been categorized as surfactin, though there has been the difference in the structural features. The major difference was observed in lipid moiety and the peptide sequence. Furthermore, the researchers have also realized the difference in the ionic nature, hemolytic behavior, and antimicrobial properties, although, the organisms displayed the gene responsible for surfactin. It has been observed that surfactin of one *Bacillus* species is entirely different from the surfactin of other *Bacillus* species. But only few reports illustrate >99% matching with the commercially available surfactin.

In the present study, though the surface active agent of *B. licheniformis* and *B. subtilis* showed similarity among them, the gene of expression clearly demonstrated the difference. *srf* expression was observed in both *B. licheniformis* and *B. subtilis*, whereas *sfp* expression was realized only with *B. subtilis*. It has been suggested that in *B. licheniformis* the integration between *srf* and *sfp *genes may not occur, which limits the expression of *sfp *gene, whereas the integration between said genes in *B. subtilis* expresses the *sfp *gene. It may also be argued that the selection of primers could be one of the reasons or the directed *de nova* synthesis may hinder the expression of *sfp*.

## 4. Conclusion

The present study deals with the discrimination in surfactin- like biosurfactant extracted from marine *Bacillus *species of Tamil coastal plain that was studied in detail and the expression of genes responsible for surface active agent demonstrated wide variations, which added an additional information for the Gram positive bacterial species of marine origin.

On comparing these observations with the reported literatures it has been concluded that *Bacillus* spp. of Tamil Nadu coastal plains have the capacity to produce biosurfactant, having similar or more or less equal surfactant activity compared to other *Bacillus* spp. but displayed nonionic and nonhemolytic behaviour. This property of the biosurfactant is interesting and needs explorations. Among the *B. licheniformis* and *B. subtilis* species, the gene specific to biosurfactant production (*sfp*) has been expressed in *B. subtilis* and not in *B. licheniformis*.

## Figures and Tables

**Table 1:** Phenotypic and biochemical characteristic of marine isolates.

Characteristics	Isolate 1	Isolate 2	Isolate 3	Isolate 4	Isolate 5
Morphology	Pink, dry, and filamentous	White, smooth, and irregular colonies	White, smooth, circular, and undulate margin	White, circular, and smooth margin	White, smooth, slimy, and entire margin
Gram stain	+	+	+	+	+
Shape	Rod	Rod	Rod	Rod	Rod
Motility	Motile	Motile	Motile	Motile	Motile
Indole	−	−	−	−	−
Methyl red	−	−	−	−	−
Voges Proskauer	+	+	+	+	+
Citrate	+	+	+	+	+
Urease	−	−	−	−	+
Nitrate	+	+	−	−	+
Oxidase	+	−	−	−	−
Catalase	+	+	+	+	+
Starch hydrolysis	+	+	+	+	+
Casein hydrolysis	+	+	+	+	+
Lipid hydrolysis	+	+	+	+	+
Gelatin hydrolysis	+	+	+	+	+
Growth at 55°C	+	+	−	−	−
Growth in NaCl					
5%	+	+	+	+	+
7.5%	+	+	+	+	+

(+): positive; (−): negative.

**Table 2:** 16S rRNA sequence homology. Identification and comparison of the bacterial isolates from marine environment.

Isolates	The best GenBank match		
	Classification (accession number)	% homology	% coverage
Isolate 1	*B. licheniformis ATCC 14580* (NC006270.3)	94%	99%

Isolate 2	*B. licheniformis DSM13 = ATCC 14580* (NC006270.1)	99%	100%

Isolate 3	*B. subtilis * RO-NN-1 chromosome (NC019948.1)	100%	100%

Isolate 4	*Bacillus* spp. JS chromosome (NC017743.1)	100%	100%

Isolate 5	*B. subtilis BEST7613 DNA* (NC019948.1)	100%	99%

**Table 3:** Surfactant activity of the 5 marine isolates.

Isolate	DCT	ODT	TM (mN/m)
Isolate 1	+++	+++	28 ± 3
Isolate 2	+++	+++	31 ± 2
Isolate 3	++	+	29 ± 4
Isolate 4	+++	+++	30 ± 2
Isolate 5	+++	+++	26 ± 4

(+++): excellent; (++): good; (+): average [DCT: drop collapse test; ODT: oil displacement test; TM: tensiometer measurement].

**Table 4:** Chemical composition of biosurfactants obtained from marine isolates.

Marine isolates	Lipid content (µg/mL)	Protein content (µg/mL)	Carbohydrate content (µg/mL)	Haemolytic activity	Antimicrobial activity	Ionic character
*Microsurf-1 *	17.5	103.3	8.61	Nonhaemolytic	No activity	Nonionic
*Microsurf-2 *	7.59	80.28	2.65	Nonhaemolytic	No activity	Nonionic
*Microsurf-3 *	69.1	16.73	0.72	Nonhaemolytic	No activity	Nonionic
*Microsurf-4 *	17.78	20.55	7.19	Nonhaemolytic	No activity	Nonionic
*Microsurf-5 *	9.7	5.39	0.235	Nonhaemolytic	No activity	Nonionic
Standard (surfactin)	—	—	—	Haemolytic	Broad spectrum	Anionic
